# MHC Genomics and Disease: Looking Back to Go Forward

**DOI:** 10.3390/cells8090944

**Published:** 2019-08-21

**Authors:** Roger L. Dawkins, Sally S. Lloyd

**Affiliations:** Centre for Innovation in Agriculture, Murdoch University and C Y O’Connor ERADE Village Foundation, North Dandalup 6207, Western Australia, Australia

**Keywords:** MHC, ancestral haplotype, autoimmune disease

## Abstract

Ancestral haplotypes are conserved but extremely polymorphic kilobase sequences, which have been faithfully inherited over at least hundreds of generations in spite of migration and admixture. They carry susceptibility and resistance to diverse diseases, including deficiencies of CYP21 hydroxylase (47.1) and complement components (18.1), as well as numerous autoimmune diseases (8.1). The haplotypes are detected by segregation within ethnic groups rather than by SNPs and GWAS. Susceptibility to some other diseases is carried by specific alleles shared by multiple ancestral haplotypes, e.g., ankylosing spondylitis and narcolepsy. The difference between these two types of association may explain the disappointment with many GWAS. Here we propose a pathway for combining the two different approaches. SNP typing is most useful after the conserved ancestral haplotypes have been defined by other methods.

## 1. Introduction

It has been very nearly 50 years since Terasaki, Brewerton, and their colleagues discovered the extraordinary association between ankylosing spondylitis (AS) and HLA B27 [1,2]. With a few caveats of great interest to clinicians, all patients with AS have this allele, justifying the idea that B27 is an essential requirement for the disease–effectively a sine qua non [3]. However, the allele is much more frequent than the disease, and is therefore not itself sufficient. Penetrance is low [4,5]. Other known requirements are male sex and adult age, indicating that the mechanisms of susceptibility and pathogenesis may be quite complex and difficult to unravel. This is still the case today, although the fundamental finding has been confirmed to exhaustion [6].

It has been nearly 40 years since it was established that other HLA associations are completely different [3,7]. For example, in Caucasoids, the more severe forms of systemic lupus erythematosus (SLE) and myasthenia gravis with thymic hyperplasia (MG) are associated with the 8.1 ancestral haplotype [8,9]. This sequence includes B8, but extends over more than a megabase from HLA A to HLA DP. The genetic factors responsible for susceptibility and severity must be numerous and widely spaced throughout the MHC, although most evidence implicates the central MHC, including the *C2*, *Bf*, *C4*, *HSP*, and *TNF* genes, together with their associated non-coding or regulatory sequences. In contrast to AS, female sex is important for these conditions [10].

By 1983, these two different types of association were well recognized [8]. Over subsequent decades, many more examples have been added. Narcolepsy is another example of the allelic form in that the DQB1 0602 association is not restricted to one ethnic group [11,12]. Therefore, explanations for a direct role of a single allele are sought, in this case, and may ultimately appear to be disarmingly simple as for haemochromatosis [13,14] and some drug hypersensitivities [15].

Deficiencies of C4, C2, and 21-hydroxylase (21OH) are examples of associations with extensive ancestral haplotypes (8.1, 18.1, and 47.1, respectively) [16,17]. In each case, there is a plausible explanation, in that the particular sequence includes a missing or defective gene. The observations by Alper et al. [7,18,19] were important in leading to concepts of conserved population haplotypes, which have been faithfully inherited over thousands of generations and are best illustrated by 57.1, which is represented to various degrees in multiple ethnicities.

**Figure 1:**
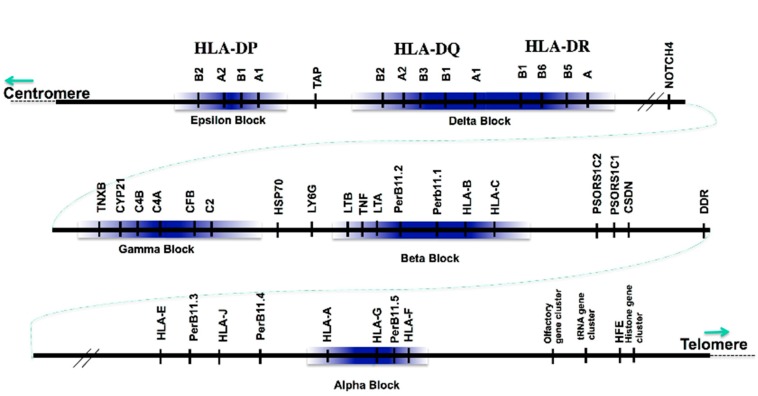
Polymorphic frozen blocks (PFB) in the MHC. Reproduced with permission from [5], and adapted from [16].

As different ancestral groups formed and migrated out of Africa and beyond, they carried conserved MHC sequences, which were fixed at each of the alpha, beta, gamma, and delta polymorphic frozen blocks (PFB) (see Figure 1 and Table 1). These sequences were shuffled laterally somewhat as populations mixed, and new combinations appeared, but the more polymorphic regions survive to this day [20,21,22]. In the case of these deficiencies, the defective or missing *C4*, *C2*, or 21OH genes remain within the frozen sequence.

The organization of the MHC provides a model for the genome. Each ancestral haplotype has its own map. Polymorphic frozen blocks are shaded. Not all genes are shown. PerB11 is now designated *MIC*. Frozen refers to the freezing of the sequence by inhibition of recombination and mutations, whether

1. unequal crossing-over,
2. double recombination,
3. nucleotide replacement,
4. insertions and deletions,
5. duplication,
6. other.

**Figure 2:**
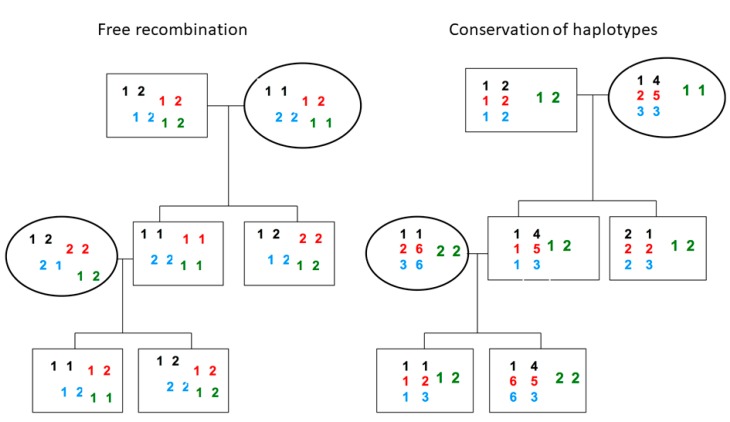
Stable segregation of ancestral haplotypes through three generation family trees. The left describes the inheritance pattern, where the loci are bi-allelic and segregate independently. The right describes the inheritance pattern within PFB, where the alleles are highly polymorphic and the four ancestral haplotypes have segregated predictably.

It was only through extensive family studies that it became clear that recombination occurred outside polymorphic frozen blocks (Figure 2). Indeed, the frozen blocks must have been inherited faithfully over many generations, since identical haplotypes occur in subjects with extensive family trees showing no known relationship, and even in populations that were widely scattered geographically, implying only very remote common ancestry.

The reality is that the frozen blocks occupy only a limited proportion of the whole MHC region of a megabase or more, and we have not been able, as suggested in Figure 1, to define hard boundaries between frozen blocks and areas subject to recombination. This difficulty leads us to conclude that there are degrees of freezing as well as specific hotspots. Carrington [23] has contributed by identifying some of the regions which do recombine, but we suspect that such regions are dependent on the genomic environment rather than distance. For example, B8, which is Caucasoid specific by any measure, occurs in non- caucasoids with MG but hardly otherwise [24]. This observation implies that lateral transfer between haplotypes of very different ancestry may lead to thawing as a consequence of differences in the cis, trans, and epistatic interactions. Using multiethnic mapping is powerful [25,26,27] as a way to find which components carry susceptibility. In other words, ancestral haplotypes are preserved in ethnicities, but eventually fall apart in multiracial combinations.

Susceptibility to autoimmune diseases, such SLE, MG, and insulin-dependent diabetes mellitus (IDDM) must be similarly frozen [8,28,29,30,31,32]—although, to date, there is no mechanistic explanation for the susceptibility [20,32]. Largely, for this reason, we have suggested a dominant role for non-coding regulatory sequences associated with duplicons, indels, and retroviral-like elements (Table 1). We also implicate *epistatic, trans*, and *cis* interactions, with their potential to increase the degree of functional polymorphism exponentially [5,16]. *Epistatic* refers to sequences on different chromosomes, which segregate independently. *Trans* refers to the sequence of the second alternative chromosome. *Cis* refers to the same chromosome.

These lessons could not have been predicted from the prevailing concepts, which still underlie the thinking behind SNPs and GWAS (Table 2). This SNP-based thinking promotes an overemphasis on the role of ongoing mutation, compared to conservation of ancient polymorphism [33].

**Figure 3:**
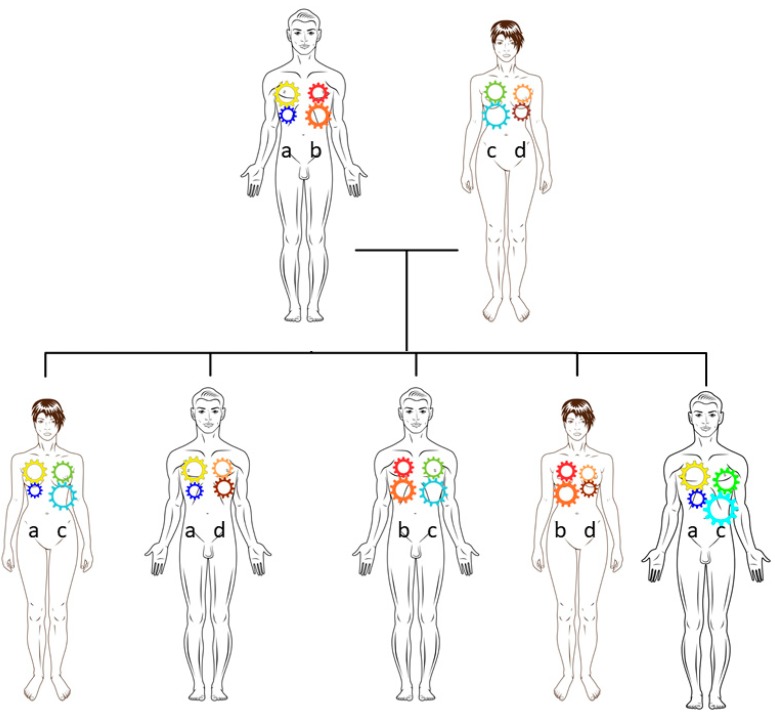
Segregating ancestral haplotypes preserve cis interactions. Ancestral haplotypes are represented by two cogs meshing vertically (in *cis*). The size and shape of cogs represent polymorphism or variant forms of the blocks. For example, the father has yellow–blue (a) and red–orange (b) haplotypes while the mother has green-cyan (c) and orange-brown (d) haplotypes. Haplotype (a) has been transmitted to the eldest daughter, eldest son, and youngest son. The middle two children have inherited haplotype (b). Reactive meshing is dependent on hormonal and other environmental influences. The oldest and youngest offspring are genotypically identical, but the interactions are different as a consequence of the sexual environment. Adapted with permission from [5].

Clearly, any understanding of MHC genomics leading to disease must take account of the pragmatic observations implicating age- and sex-dependent penetrance. In Figure 3, we illustrate the potential importance of cis and trans interactions by proposing that ancestral haplotypes can be represented by meshing polymorphic cogs. The degree of meshing or interaction, whether cis or trans, is affected by size and density (expression), as well as by competition with similar cogs, including those representing paralogous sequences encoded on other chromosomes. Thus, MHC, genotypically identical subjects may be affected to different degrees (Figure 3).

These models have led to the concept of multifunctional polymorphic control of metabolic and other pathways. Cascades involving stepwise activation of related products, such as the complement system, are promising targets for further study.

## 2. Recognition of Conserved Ancestral Haplotypes

Table 3 illustrates how ancestral haplotypes were recognized initially. Today there are many more haplotypic markers, including noncoding sequences between the loci shown. As the number of haplotypic markers increased, including SNPs, it became more and more obvious that very few are haplo-specific. It follows that linkage disequilibrium (LD) cannot define such haplotypes. For example, 2 × 2 delta values cannot identify 18.1 and 18.2, because the alleles are shared by other haplotypes.

## 3. Genome-Wide Association Studies and Single Nucleotide Polymorphisms

We believe that the above concepts and models of ancestral haplotypes suggest an alternative approach to the conduct and analysis for genome-wide association studies. When applied to the MHC, some results of commercial SNP typing have been disappointing, even to the point that a recent review by Kennedy et al. [36] essentially dismisses the many classic studies, and very unwisely blames “HLA typing errors, disregard of population structure and lack of replication” [36]. The same authors cite a paper which promotes a “focus on haplotypes … first suggested in 1987”, thereby ignoring important previous contributions on haplotypes—including the original use of the term in 1967 by Ruggero Ceppellini [37,38]—and a huge body of careful observations that have been confirmed repeatedly and rediscovered, without attribution, in the past few decades.

The International Hapmap Project [39,40,41] is a potentially valuable resource of high-resolution SNP-typed individuals, and includes samples of cell lines used to define the genomic sequence of conserved, extended ancestral haplotypes, which segregate faithfully through families. Surprisingly, the proponents and users [42] of Hapmap have ignored the opportunities for reconciliation with earlier studies, which addressed the shortcomings of LD analysis and focused on haplotypic sequences, including RLEs; indels; duplications; and single nucleotide polymorphism in the literal sense, used by Gaudieri et al. [43] and Longman et al. [44] to map regions of extensive, interrupted sequence differences. The importance of PFB [16] or fixity [45] was also ignored until rediscovered [46].

A more balanced review by Petersdorf and O’hUigin [47] begins the daunting process of integrating population genetics, classic HLA associations, ancestral haplotypes, polymorphic frozen blocks, SNP typing, and gene expression [47]. The authors hope for “the study of haplotype-associated phenotypic differences” and for “haplotype-matching” in transplantation. Indeed, there is already great encouragement for each of these ambitions. The functional differences conferred by ancestral haplotypes, such as 8.1, have been well known for more than 30 years, and include *TNF* and *IgA* concentrations, even though the latter is not encoded within the MHC [16,48,49]. The benefit of haplotype matching in renal and bone marrow transplantation was established decades ago [50,51].

## 4. Reconciling MHC Genomics and SNP Typing

While we recognize that the disconnect between classical MHC and later SNP genomics will decrease [47,52], we hope this happens quickly. To this end, we summarize the issues as follows:

1. There is no possibility of a single reference sequence. Rather, there are numerous ancestral haplotypes, each with its own very extensive and specific sequence [35,53,54,55].
2. These sequences are characteristic of ancestral populations or ethnicities. SNP typing on heterozygous mixed populations cannot reveal ancestral haplotype—or at least, must be extremely inefficient [20].
3. MHC complexity is best managed by defining haplotypes through segregation in extensive family studies [56]—not trios—since the power of segregation increases with the number of copies in different heterozygous combinations. Recombination can be demonstrated given sufficient generations to study.
4. Ancestral haplotypes include specific duplications, indels, etc. [54]. Fortunately, there are now many panels of homozygous cell lines and libraries of their sequences available [57,58]. These should be the references and should replace allele and SNP databases.
5. Penetrance is crucial but complex, and depends on age, sex, and a multitude of environmental factors that will vary from time to time and in different settings [53]. Cis and trans interactions are well known contributors to susceptibility and severity [5] These need to be included in the experimental design, but will be difficult to understand until the relevant pathways are defined. We recommend careful consideration of the concept of whole genome duplications resulting in paralogous sequences [16,59], which may compete with and modify the effect of any sequence implicated through SNP analysis.
6. Linkage disequilibrium (LD) is an incomplete reflection of conserved haplotypes and their relative frequencies. Many highly conserved haplotypes cannot be detected by delta values, because even though haplotypic (i.e., present on all examples), individual SNPs are *not* haplo-specific. Different haplotypes share nucleotides and alleles at different locations and relative frequencies. LD between SNPs is misleading if used to define functional haplotypes. Haplospecific recombination creates further complexity [23,60,61].
7. There are at least two types of association with disease, as described above. No doubt, there will be further categories, especially as cis and trans interactions are defined. IDDM is an example of the need to address the mode of inheritance and multiple interactions, as described by Alper et al. [62,63]. Epistatic interactions may also be important.
8. The low positive predictive values of a genetic marker for a disease will remain so until the pathogenic pathways are understood. Fortunately, for clinical purposes, there are examples where the absence of an allele or sequence can be useful for the exclusion of a diagnosis. However, the presence of the same allele does not permit confirmation of the diagnosis—take, for example, HLA B27 [4]. Thus, those designing future studies should consider how the results will be of practical value, and at the same time, be useful in defining the biology.
9. Many regions of biological and statistical importance are not included in commercial SNP typing. In fact, duplications, indels, RLEs, and ambiguities may be very informative [16].
10. The MHC is a useful model for other genomic regions with polymorphic frozen blocks [64].
11. An understanding of synteny and paralogy is very valuable [65].
12. Many MHC associations, such as narcolepsy, 21-OH deficiency, and haemochromatosis are *not* immunologically mediated. There is no justification for prejudice in interpreting results. Current limited understanding of pathological processes may lead to confusion. In fact, as illustrated by the value of informative clinic-to-laboratory studies, there is potential to elucidate these processes.
13. A promising approach includes understanding the processes and genetics responsible for autoimmune diseases induced by immune checkpoint inhibitors, vaccines, and drugs, such as D-Penicillamine. The value of genomics increases when the inducing agent is known [15].

## 5. Conclusions

One lesson might be that there are incompatible concepts and terminology, as well as a very patchy understanding of the incontrovertible facts of MHC associations and structure. Another might be that research is constrained by the limitations of commercial platforms.

We hope our commentary is helpful in providing background for new discoveries. Hence the subtitle: “Looking Back to Go Forward”. Surely, it is important not to dismiss the history as “HLA typing errors”. In fact, the International HLA workshops provide a useful resource for haplotype association with disease, as well as for matching donors and hosts for transplantation.

SNP typing is most useful after the conserved ancestral haplotypes have been defined by other methods.

## Figures and Tables

**Table 1:** Lessons from MHC genomics.

• Human diversity is inherited from ancestors, rather than created by recent mutation.
• Diversity is regenerated at speciation and maintained by meiotic recombination between the ancestral haplotypes within polymorphic frozen blocks.
• The unit of inheritance is the ancestral haplotype.
• Such sequences carry specific alleles, duplicons, indels and retroviral-like elements (RLEs), which together regulate gene expression.

Reproduced with permission from [5].

**Table 2:** Alternative dogma.

• Genetic diversity or inherited variation requires ongoing mutation.
• Diversity accumulates through errors in the copying of DNA.
• The unit of inheritance is the allele.
• At each locus alleles may be deleterious, beneficial, or neutral.
• Meiotic recombination scrambles maternal and paternal alleles.
• Linkage disequilibrium between SNPs defines haplotypes.

Reproduced with permission from [5].

**Table 3:** Haplotype definitions and frequencies in an Australian population.

Ancestral Haplotype	A	Cw	B	C2	Bf	C4A	C4B	DR	DQ	Frequency
7.1	3	7	7	C	S	3	1	2	6	12.9%
8.1	1	7	8	C	S	0	1	3	2	13.2%
13.1			13		S	3	1	7		2.6%
18.1	25	-	18	0	S	4	2	3	6	1.1%
18.2	30	5	18	C	F1	3	0	3	2	1.7%
18.3			18		S	3	1	5		5.2%
35.1		4	35		S	3	1	5		6.9%
35.2	3	4	35	C	F	3 + 2	0	1	5	0.9%
35.3	11	4	35		S	3	0	1	5	2.3%
44.1	2	5	44	C	S	3	0	4	7	5.5%
44.2	29	4	44	C	F	3	1	7	2	2.6%
47.1	3	6	47	C	F	1	0	7	2	<0.6%
57.1	1	6	57	C	S	6	1	7	9	2.6%
65.1		8	65	C	S	2	1 + 2	1	5	0.6%

Adapted from [34,35].

## References

[1] Brewerton D.A., Hart F.D., Nicholls A., Caffrey M., James D.C.O., Sturrock R.D. (1973). Ankylosing Spondylitis and HL-A 27. Lancet.

[2] Schlosstein L., Terasaki P.I., Bluestone R., Pearson C.M. (1973). High association of an HL-A antigen, W27, with ankylosing spondylitis. N. Engl. J. Med..

[3] Dawkins R.L., Christiansen F.T., Zilko P.J. Musculoskeletal Disease and D-penicillamine, including reports from the D-Pen-HLA 82 Workshop. Immunogenet. Rheumatol..

[4] Christiansen F.T., Hawkins B.R., Dawkins R.L., Owen E.T., Potter R.M. (1979). The prevalence of ankylosing spondylitis among B27 positive normal individuals—A reassessment. J. Rheumatol..

[5] Dawkins R.L. (2015). Adapting Genetics.

[6] Brown M.A., Kenna T., Wordsworth B.P. (2016). Genetics of ankylosing spondylitis—Insights into pathogenesis. Nat. Rev. Rheumatol..

[7] Alper C.A., Awdeh Z.L., Raum D.D., Yunis E.J. (1982). Extended major histocompatibility complex haplotypes in man: Role of alleles analogous to murine t mutants. Clin. Immunol. Immunopathol..

[8] Dawkins R.L., Christiansen F.T., Kay P.H., Garlepp M., McCluskey J., Hollingsworth P.N., Zilko P.J. (1983). Disease associations with complotypes, supratypes and haplotypes. Immunol. Rev..

[9] Rigby R.J., Dawkins R.L., Wetherall J.D., Hawkins B.R. (1978). HLA in systemic lupus erythematosus: Influence on severity. Tissue Antigens.

[10] Christiansen F.T., Dawkins R.L., Uko G., McCluskey J., Kay P.H., Zilko P.J. (1983). Complement allotyping in SLE: association with C4A null. Aust. N. Z. J. Med..

[11] Chabas D., Taheri S., Renier C., Mignot E. (2003). The Genetics of Narcolepsy. Annu. Rev. Genom. Hum. Genet..

[12] Hollingsworth P.N., Dawkins R.L., Peter J.B. (1993). HLA and narcolepsy. Neurology.

[13] Gerhard G.S., Ten Elshof A.E., Chorney M.J. (1998). Hereditary haemochromatosis as an immunological disease. Br. J. Haematol..

[14] Feder J.N., Gnirke A., Thomas W., Tsuchihashi Z., Ruddy D.A., Basava A., Dormishian F., Domingo R., Ellis M.C., Fullan A. (1996). A novel MHC class I-like gene is mutated in patients with hereditary haemochromatosis. Nat. Genet..

[15] Mallal S., Nolan D., Witt C., Masel G., Martin A.M., Moore C., Sayer D., Castley A., Mamotte C., Maxwell D. (2002). Association between presence of HLA-B*5701, HLA-DR7, and HLA-DQ3 and hypersensitivity to HIV-1 reverse-transcriptase inhibitor abacavir. Lancet.

[16] Dawkins R.L., Leelayuwat C., Gaudieri S., Tay G., Hui J., Cattley S., Martinez P., Kulski J. (1999). Genomics of the major histocompatibility complex: haplotypes, duplication, retroviruses and disease. Immunol. Rev..

[17] McCluskey J., Kay P.H., Stickey M., Christiansen F.T., Dawkins R.L., Wilson G. (1983). MHC “supratype” predicting heterozygous 21-hydroxylase deficiency. Lancet.

[18] White P.C., New M.I., Dupont B. (1984). HLA-linked congenital adrenal hyperplasia results from a defective gene encoding a cytochrome P-450 specific for steroid 21-hydroxylation. Proc. Natl. Acad. Sci. USA.

[19] Raum D., Awdeh Z., Yunis E.J., Alper C.A., Gabbay K.H. (1984). Extended major histocompatibility complex haplotypes in type I diabetes mellitus. J. Clin. Investig..

[20] Alper C.A., Larsen C.E. (2015). Major Histocompatibility Complex: Disease Associations. eLS.

[21] Gaudieri S., Leelayuwat C., Tay G.K., Townend D.C., Dawkins R.L. (1997). The major histocompatibility complex (MHC) contains conserved polymorphic genomic sequences that are shuffled by recombination to form ethnic-specific haplotypes. J. Mol. Evol..

[22] Lam T.H., Tay M.Z., Wang B., Xiao Z., Ren E.C. (2015). Intrahaplotypic variants differentiate complex linkage disequilibrium within human MHC haplotypes. Sci. Rep..

[23] Carrington M. (1999). Recombination within the human MHC. Immunol. Rev..

[24] Christiansen F.T., Pollack M.S., Garlepp M.J., Dawkins R.L. (1984). Myasthenia gravis and HLA antigens in American blacks and other races. J. Neuroimmunol..

[25] Todd J.A., Mijovic C., Fletcher J., Jenkins D., Bradwell A.R., Barnett A.H. (1989). Identification of susceptibility loci for insulin-dependent diabetes mellitus by trans-racial gene mapping. Nature.

[26] Alper C.A., Larsen C.E., Dubey D.P., Awdeh Z.L., Fici D.A., Yunis E.J. (2006). The Haplotype Structure of the Human Major Histocompatibility Complex. Hum. Immunol..

[27] Degli-Esposti M.A., Andreas A., Christiansen F.T., Schalke B., Albert E., Dawkins R.L. (1992). An approach to the localization of the susceptibility genes for generalized myasthenia gravis by mapping recombinant ancestral haplotypes. Immunogenetics.

[28] Kelly H., McCann V.J., Kay P.H., Dawkins R.L. (1985). Susceptibility to IDDM is marked by MHC supratypes rather than individual alleles. Immunogenetics.

[29] Fernando M.M., Stevens C.R., Sabeti P.C., Walsh E.C., McWhinnie A.J., Shah A., Green T., Rioux J.D., Vyse T.J. (2007). Identification of two independent risk factors for lupus within the MHC in United Kingdom families. PLoS Genet..

[30] Pugliese A., Gianani R., Moromisato R., Awdeh Z.L., Alper C.A., Erlich H.A., Jackson R.A., Eisenbarth G.S. (1995). HLA-DQB1*0602 is associated with dominant protection from diabetes even among islet cell antibody-positive first-degree relatives of patients with IDDM. Diabetes.

[31] Schloot N.C., Roep B.O., Wegmann D., Yu L., Chase H.P., Wang T., Eisenbarth G.S. (1997). Altered immune response to insulin in newly diagnosed compared to insulin-treated diabetic patients and healthy control subjects. Diabetologia.

[32] Rewers M., Norris J.M., Eisenbarth G.S., Erlich H.A., Beaty B., Klingensmith G., Hoffman M., Yu L., Bugawan T.L., Blair A. (1996). Beta-cell autoantibodies in infants and toddlers without IDDM relatives: Diabetes autoimmunity study in the young (DAISY). J. Autoimmun..

[33] Dawkins R.L., Willamson J.F., Lester S., Dawkins S.T. (2013). Mutation versus polymorphism in evolution. Genomics.

[34] Dawkins R.L., Leaver A., Cameron P.U., Martin E., Kay P.H., Christiansen F.T. (1989). Some disease-associated ancestral haplotypes carry a polymorphism of TNF. Hum. Immunol..

[35] Degli-Esposti M.A., Leaver A.L., Frank T., Witt C.S., Abraham L.J., Dawkins R.L. (1992). Ancestral Haplotypes: Conserved Population MHC Haplotypes. Hum. Immunol..

[36] Kennedy A.E., Ozbek U., Dorak M.T. (2017). What has GWAS done for HLA and disease associations?. Int. J. Immunogenet..

[37] Ceppellini R., Curtoni E.S., Mattuiz P.L., Miggiano V., Scudeller G., Serra A., Curtoni E.S., Mattiuz P.L., Tosi R.M. (1967). Genetics of Leukocyte Antigens: A Family Study of Segregation and Linkage. Histocompatibility Testing 1967.

[38] Petersdorf E.W. (2017). In celebration of Ruggero Ceppellini: HLA in transplantation. HLA.

[39] Manolio T.A., Brooks L.D., Collins F.S. (2008). A HapMap harvest of insights into the genetics of common disease. Fournal Clin. Investig..

[40] International HapMap Consortium (2005). A haplotype map of the human genome. Nature.

[41] International HapMap Consortium (2003). The International HapMap Project. Nature.

[42] Ka S., Lee S., Hong J., Cho Y., Sung J., Kim H.N., Kim H.L., Jung J. (2017). HLAscan: Genotyping of the HLA region using next-generation sequencing data. BMC Bioinform..

[43] Gaudieri S., Dawkins R.L., Habara K., Kulski J.K., Gojobori T. (2000). SNP profile within the Human Major Histocompatibility Complex reveals an extreme and interrupted level of nucleotide diversity. Genome Res..

[44] Longman-Jacobsen N., Williamson J.F., Dawkins R.L., Gaudieri S. (2003). In Polymorphic Genomic Regions Indels Cluster with Nucleotide Polymorphism: Quantum Genomics. Gene.

[45] Romero V., Larsen C.E., Duke-Cohan J.S., Fox E.A., Romero T., Clavijo O.P., Fici D.A., Husain Z., Almeciga I., Alford D.R. (2007). Genetic fixity in the human major histocompatibility complex and block size diversity in the class I region including HLA-E. BMC Genet..

[46] Gabriel S.B., Schaffner S.F., Nguyen H., Moore J.M., Blumenstiel B., Higgins J., Defelice M., Lochner A., Faggart M., Liu-cordero S.N. (2002). The Structure of Haplotype Blocks in the Human Genome. Science.

[47] Petersdorf E.W., O’hUigin C. (2019). The MHC in the era of next-generation sequencing: Implications for bridging structure with function. Hum. Immunol..

[48] Abraham L.J., French M.A., Dawkins R.L. (1993). Polymorphic MHC ancestral haplotypes affect the activity of tumour necrosis factor-alpha. Clin. Exp. Immunol..

[49] Wilton A.N., Cobain T.J., Dawkins R.L. (1985). Family studies of IgA deficiency. Immunogenetics.

[50] Tay G.K., Witt C.S., Christiansen F.T., Charron D., Baker D., Herrmann R., Smith L.K., Diepeveen D., Mallal S., McCluskey J. (1995). Matching for MHC haplotypes results in improved survival following unrelated bone marrow transplantation. Bone Marrow Transplant..

[51] Wilton A.N., Christiansen F.T., Dawkins R.L. (1985). Supratype matching improves renal transplantation survival. Transplant. Proc..

[52] Vadva Z., Larsen C.E., Propp B.E., Trautwein M.R., Alford D.R., Alper C.A. (2019). A New Pedigree-Based SNP Haplotype Method for Genomic Polymorphism and Genetic Studies. Cells.

[53] Lloyd S.S., Steele E.J., Dawkins R.L., Kulski J.K. (2016). Analysis of Haplotype Sequences. Next Generation Sequencing—Advances, Applications and Challenges.

[54] Horton R., Gibson R., Coggill P., Miretti M., Allcock R.J., Almeida J., Forbes S., Gilbert J.G.R., Halls K., Harrow J.L. (2008). Variation analysis and gene annotation of eight MHC haplotypes: the MHC Haplotype Project. Immunogenetics.

[55] Jensen J.M., Villesen P., Friborg R.M., Mailund T., Besenbacher S., Schierup M.H. (2017). Assembly and analysis of 100 full MHC haplotypes from the Danish population. Genome Res..

[56] Alper C.A., Larsen C.E. (2008). Pedigree-Defined Haplotypes and Their Applications to Genetic Studies. Haplotyping: Methods and Protocols, Methods in Molecular Biology.

[57] International Histocompatibility Working Group. http://www.ihwg.org/.

[58] Norman P.J., Norberg S.J., Guethlein L.A., Nemat-Gorgani N., Royce T., Wroblewski E.E., Dunn T., Mann T., Alicata C., Hollenbach J.A. (2017). Sequences of 95 human MHC haplotypes reveal extreme coding variation in genes other than highly polymorphic HLA class i and II. Genome Res..

[59] Kasahara M. (1999). The chromosomal duplication model of the major histocompatibility complex. Immunol. Rev..

[60] van Oosterhout C. (2009). Trans-species polymorphism, HLA-disease associations and the evolution of the MHC. Commun. Integr. Biol..

[61] Ahmad T., Neville M., Marshall S.E., Armuzzi A., Mulcahy-Hawes K., Crawshaw J., Sato H., Ling K.L., Barnardo M., Goldthorpe S. (2003). Haplotype-specific linkage disequilibrium patterns define the genetic topography of the human MHC. Hum. Mol. Genet..

[62] Aly T.A., Eller E., Ide A., Gowan K., Babu S.R., Erlich H.A., Rewers M.J., Eisenbarth G.S., Fain P.R. (2006). Multi-SNP analysis of MHC region: remarkable conservation of HLA-A1-B8-DR3 haplotype. Diabetes.

[63] Hutton J.C., Eisenbarth G.S. (2003). A pancreatic β-cell-specific homolog of glucose-6-phosphatase emerges as a major target of cell-mediated autoimmunity in diabetes. Proc. Natl. Acad. Sci. USA.

[64] McLure C.A., Hinchliffe P., Lester S., Williamson J.F., Millman J.A., Keating P.J., Stewart B.J., Dawkins R.L. (2013). Genomic evolution and polymorphism: Segmental duplications and haplotypes at 108 regions on 21 chromosomes. Genomics.

[65] Dawkins R.L., Berry J., Martinez P., Gaudieri S., Hui J., Cattley S., Longman N., Kulski J., Carnegie P., Kasahara M. (2000). Potential for Paralogous Mapping to Simplify the Genetics of Diseases and Functions Associated with MHC Haplotypes. The Major Histocompatibility Complex: Evolution, Structure, and Function.

